# Prognostic factors for head and neck cancer of unknown primary including the impact of human papilloma virus infection

**DOI:** 10.1186/s40463-017-0223-1

**Published:** 2017-06-10

**Authors:** Lars Axelsson, Jan Nyman, Hedda Haugen-Cange, Mogens Bove, Leif Johansson, Shahin De Lara, Anikó Kovács, Eva Hammerlid

**Affiliations:** 1 Department of Otorhinolaryngology - Head and Neck Surgery, University of Gothenburg, Sahlgrenska University Hospital, Göteborg, Sweden; 2 Department of Oncology, Sahlgrenska University Hospital, Göteborg, Sweden; 3 Department of Otorhinolaryngology, Norra Älvsborgs Hospital, Trollhättan, Sweden; 4 Department of Otorhinolaryngology, Central Hospital Skövde, Skövde, Sweden; 5 Department of Clinical Pathology and Genetics, Sahlgrenska University Hospital, Göteborg, Sweden

**Keywords:** Head and neck cancer, Unknown primary, Human papilloma virus, p16, Prognostic factors, Treatment

## Abstract

**Background:**

Head and neck cancer of unknown primary (HNCUP) is rare and prospective studies are lacking. The impact of different prognostic factors such as age and N stage is not completely known, the optimal treatment is not yet established, and the reported survival rates vary. In the last decade, human papilloma virus (HPV) has been identified as a common cause of and important prognostic factor in oropharyngeal cancer, and there is now growing interest in the importance of HPV for HNCUP. The aim of the present study on curatively treated HNCUP was to investigate the prognostic importance of different factors, including HPV status, treatment, and overall survival.

**Methods:**

A search for HNCUP was performed in the Swedish Cancer Registry, Western health district, between the years 1992–2009. The medical records were reviewed, and only patients with squamous cell carcinoma or undifferentiated carcinoma treated with curative intent were included. The tumor specimens were retrospectively analyzed for HPV with p16 immunostaining.

**Results:**

Sixty-eight patients were included. The mean age was 59 years. The majority were males, and had N2 tumors. Sixty-nine percent of the tumors were HPV positive using p16 staining. Patients who were older than 70 years, patients with N3-stage tumors, and patients with tumors that were p16 negative had a significantly worse prognosis. The overall 5-year survival rate for patients with p16-positive tumors was 88% vs 61% for p16-negative tumors. Treatment with neck dissection and postoperative radiation or (chemo) radiation had 81 and 88% 5-year survival rates, respectively. The overall and disease-free 5-year survival rates for all patients in the study were 82 and 74%.

**Conclusions:**

Curatively treated HNCUP had good survival. HPV infection was common. Independent prognostic factors for survival were age over 70 years, HPV status and N3 stage. We recommend that HPV analysis should be performed routinely for HNCUP. Treatment with neck dissection and postoperative radiation or (chemo) radiation showed similar survival rates.

## Background

Head and neck cancer of unknown primary (HNCUP) is rare, with an incidence of 0.47 per 100,000/year in Sweden [1]. The diagnostic investigation of HNCUP is extensive and aims to find the primary tumor, and the diagnosis is used when no primary tumor is found.

The prognostic importance of different clinical factors in HNCUP has previously been studied [2–6]. Low age has been associated with improved survival [2, 3]. The importance of N stage has varied in reports as some authors have found statistically worse survival for N3, others for N2b, N2c and N3, and others decreasing for N1, N2 and N3 [2, 4, 7, 8]. Extracapsular extension (ECE) of the tumor has been a negative prognostic factor [3, 8]. Smoking and alcohol overconsumption are well-known causal factors for head and neck (HN) cancer [9], and in recent years, human papilloma virus (HPV) has been identified in a significant proportion of HN cancers, especially tonsil cancer [10]. Recently, there has been a growing interest in examining the prevalence and prognostic importance of HPV in HNCUP, but no change in diagnostic recommendations or treatment guidelines has been established [11, 12].

The optimal treatment for HNCUP has not been decided, and today, the recommendations vary between different cancer centers. The most common treatments are either neck dissection (ND) and postoperative radiation or primary (chemo) radiation. No randomized treatment study has been performed for HNCUP, but several retrospective studies have reported different results [8, 13, 14]. The reported overall survival rate for HNCUP has differed greatly, with 5-year survival rates ranging from 22 to 89% [5, 15–17].

The aim of the present study of curatively treated HNCUP was to investigate the prognostic importance of different prognostic factors, including HPV status, treatment, and overall survival.

## Methods

### Study design

Data were collected from the Swedish Cancer Registry for all patients who were initially diagnosed with HNCUP, International Classification of Diseases (ICD)-10, C770, in the Western Health District from 1993 to 2009. One hundred ninety-six patients were identified; however, 111 patients were excluded after being diagnosed with a primary cancer or due to histological diagnoses other than squamous cell or undifferentiated carcinomas (e.g., adenocarcinoma, malignant melanoma, lymphoma, and salivary gland cancer). Of the remaining 85 patients, another eight did not complete the medical work-up, and nine had palliative intended treatment and were excluded, making the study population 68.

### Diagnostic work-up

All patients were examined by a specialist in Otolaryngology–Head and Neck Surgery. A fine-needle aspiration was performed from the neck mass, which provided a definitive diagnosis in 35% of the cases, whereas in 65%, an open biopsy was needed. PET-CT was performed in 32% of the patients, MRI was performed in 77% of the patients, and in 10% a CT scan was the only radiological examination. The thorax was also radiologically examined in all patients (74% CT thorax and 26% CXR). In all patients, a panendoscopy was performed, including examination of all parts of the pharynx, larynx, lungs and esophagus, as well as a tonsillectomy (31% bilateral, 57% ipsilateral, and 12% tonsillectomy during childhood). In 60%, biopsies were taken from the base of the tongue, and in 79%, biopsies were taken from the nasopharynx. All the patients were discussed by a multidisciplinary tumor board for treatment decisions. Tumor stage was based on clinical findings and imaging. The ECE status was based on pathologic reports only, from open biopsy or neck dissection.

### Immunohistochemistry

The histopathological specimens were retrospectively analyzed for HPV status with p16 immunostaining using light microscopy by pathologists blinded for clinical data and outcomes.

Formalin-fixed and paraffin-embedded blocks were used to prepare 4-μm-thick sections applied onto positively charged slides (Flex IHC Microscope Slides, Ref K8020, DAKO). Subsequently, the tissue sections were subjected to deparaffinization and rehydration followed by heat-induced epitope retrieval (HIER) Tris/EDTA buffer (pH 9.0) for 20 min at 97 °C using PT Link instrument (PT Link, Dakocytomation, DAKO). The tissue sections were immunostained with the p16 (CINtec Histology kit, Ref 9511, Roche) mouse monoclonal antibody (clone E6H4) using DAKO visualization system (Envision Flex High pH, Link, Ref 8000, DAKO) and DAKO stainer for IHC (Autostiner Plus, Dakocytomation, Denmark) following the manufacturer’s instruction. Peroxidase-catalyzed diaminobensidine tetrahydrochloride was used as the DAB+ chromogen to determine protein expression levels in tumors from HNCUP and then the slides were counterstained with hematoxylin. The stained slides were rinsed with deionized water followed by the dehydration process in ethanol 70%, ethanol 95%, absolute ethanol, cleared in xylene and added cover glass (Coverslipper, DAKO).

P16 was interpreted as positive if more than 5% of tumor cells showed brown nuclear or nuclear and cytoplasmic staining [18, 19]. The lowest positive value in this study was 20%.

### Treatment

The patients underwent one of three possible treatment options: Treatment A = neck dissection and postoperative radiation, Treatment B = primary radiation with or without chemotherapy, or Treatment C = neck dissection only. There was a change in treatment policy at our institution in 2004 from Treatment B to Treatment A as the primary choice.

Three different neck dissections were performed: radical neck dissection (levels 1–5), modified neck dissection (preserving one or more of the spinal accessory nerve, internal jugular vein, and sternomastoid muscle) or selective neck dissection (levels 1–3).

Radiotherapy (RT) was given in full doses of 64.6 to 68.0 Gy according to three different fractionation schedules due to the institution’s policy over time for head and neck cancer. Schedule I consisted of a hyperfractionated, accelerated RT with two daily fractions of 1.7 Gy 5 days/week for a total dose of 64.6 Gy with a 1-week break at 40.8 Gy. Schedule II was slightly accelerated RT given as 2 Gy per fraction, 6 fractions/week, to a total dose of 68 Gy. Schedule III was conventionally fractionated RT with one daily fraction of 2.0 Gy 5 days/week to a total of 66 Gy. Forty patients were treated with 3D conformal RT, and 21 patients with intensity-modulated radiotherapy (IMRT). There were no differences in radiotherapy techniques and dose volumes whether or not the neck dissection was performed. Volumes treated to full dose were the oropharynx and hypopharynx and the ipsilateral neck (level Ib-IV) if tumor stage was N1–N2b, and an adjuvant volume was used for the contralateral neck to 41–50 Gy. If the N-stage was higher, bilateral full dose irradiation was used. If there were retropharyngeal nodes or nodes in level V, the risk for primary tumor in nasopharynx was considered higher, and nasopharynx was also included in the full dose volume (13 of 61 patients, 21%, see Table 2). The irradiated volumes did not change over time or if radiotherapy was preceded by surgery.

All chemotherapy was given as induction therapy with two cycles of cisplatin (100 mg/m^2^) and 5-fluorouracil (1000 mg/m^2^ for 5 days) according to the institution’s policy at the time.

### Follow-up

All patients were followed every 3 months during the first 2 years and every 6 months thereafter for 5 years. A CT scan or MRI was performed 3 months after RT was completed and repeated annually in most patients.

### Statistical methods

The results are presented as the mean, standard deviation, median, minimum and maximum for continuous variables and as numbers and percentages for categorical variables. For comparisons between two groups, a Mann-Whitney *U* test was used for continuous variables, a Mantel-Haenszel chi-squared test for ordered categorical variables, a Chi^2^-test for non-ordered categorical variables and Fisher’s exact test for dichotomous variables. Survival analysis was performed to analyze time to death and tumor recurrence. A Kaplan-Meier plot was used to describe mortality for the study group and for subgroups. Comparisons of mortality between subgroups were analyzed with a log-rank test for dichotomous and non-ordered categorical variables and with a log-rank test for trend for ordered categorical variables. Standardized mortality rate (SMR) was used to analyze survival adjusted for age (Statistics Sweden 2016 was used as reference population), *P*-value for comparison between SMR results was estimated by Monte Carlo methods.

A forward stepwise Cox proportional hazard regression analysis was used to select independent predictors for time to death. All significance tests were two-tailed and conducted at the 5% significance level. SAS, System Version 9.4 (SAS Institute, Inc, Cary, NC, USA), was used for all statistical analyses.

## Results

### Characteristics of the study population

Sixty-eight patients curatively treated for HNCUP were studied, Table 1. The mean age was 59 years. The majority were males, 81%. Smoking status was not analyzed due to the lack of reliable data. Most patients had N2 tumors (63%), while 19% had N1 and 18% had N3 tumors. The histology of the tumors were squamous cell carcinoma (85%) or undifferentiated carcinoma (15%). Extracapsular tumor extension was seen in 27%, but with 40% missing data. A majority were p16 positive, 69%.

**Table 1:** Patient, tumor, and treatment data for all patients divided by HPV status

	All patients	p16 positive	p16 negative	*p*-value
Subjects, *n* (%)	68	41^a^ (69)	18^a^ (31)	
Age at diagnosis (years)				
Mean (SD)	59.4 (10.9)	57.2 (10.2)	63.4 (11.2)	0.082
Median (range)	58 (36–87)	56 (36–85)	62 (48–85)	
Gender, male *n* (%)	55 (81)	32 (78)	16 (89)	0.55
Histology				
SCC	58 (85)	37 (90)	13 (72)	
SCC poorly diff	30 (44)	20 (49)	9 (47)	
SCC mod-highly diff	11 (16)	6 (15)	2 (11)	
SCC not spec	17 (25)	11 (27)	2 (11)	
Carcinoma not spec	10 (15)	4 (10)	5 (28)	0.24
Extracapsular extension				
Yes	11 (16)	7 (23)	4 (44)	
No	30 (44)	23 (77)	5 (56)	0.41
N stage				
N1	13 (19)	9 (22)	1 (6)	
N2	43 (63)	24 (59)	14 (78)	
N2a	24 (35)	14 (34)	10 (56)	
N2b	13 (19)	8 (20)	2 (11)	
N2c	6 (9)	2 (5)	2 (11)	
N3	12 (18)	8 (20)	3 (17)	0.76
Treatment				
Treatment A	36 (53)	28 (68)	8 (44)	
Treatment B	25 (37)	10 (24)	6 (33)	
Treatment C	7 (10)	3 (7)	4 (22)	0.052

*HPV* human papilloma virus, *SCC* squamous cell carcinoma. Treatment A, neck dissection and postoperative (chemo) radiation. Treatment B, (chemo) radiation. Treatment C, neck dissection. ^a^In nine of 68 patients p16 analysis was not possible

### Survival

**Fig. 1:**
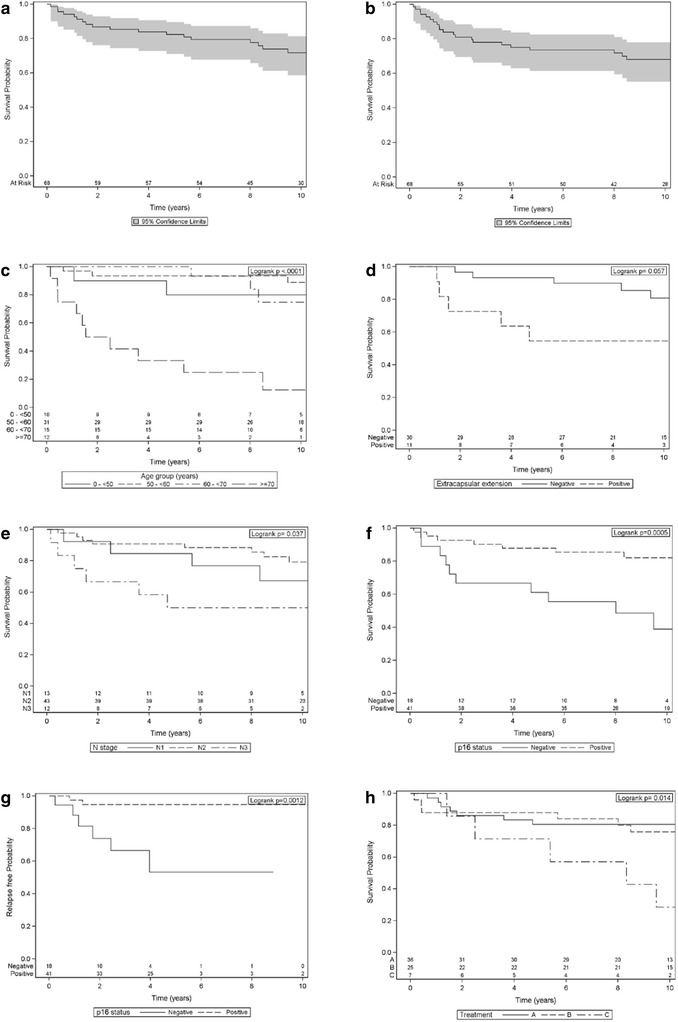
**a**–**h** Kaplan-Meier plots for different prognostic factors. The overall survival is shown in **a**, **c**, **d**, **e**, **f** and **h**, in **b** the disease-free survival, and in **g** the relapse-free probability. The number of patients at risk is shown at the bottom of the figures. Significance levels were calculated with a log-rank test. **a**, **b** The whole study population, the shaded area shows the 95% confidence limits. **c** Age groups. **d** N stage. **e** Extracapsular tumor extension. **f**, **g** p16 status. **h** Treatment. Treatment A = neck dissection and postoperative radiation, treatment B = (chemo) radiation, and treatment C = neck dissection

The overall survival rates after 2, 5 and 10 years for all patients were 87, 82, and 72%, respectively, Fig. 1a. The disease-free survival rates after 2, 5 and 10 years for all patients were 81, 74, and 68%, respectively, Fig. 1b. Among the patients who were deceased within 5 years from diagnosis (12 patients), one died due to the treatment, two from the tumor, and the remaining nine from other diseases. In total, eight patients (12%) had a recurrence of the tumor. One patient had primary tumor emergence at the base of the tongue, six patients (9%) had a local recurrence in the neck, and one patient had a distant metastasis in the bone tissue of the left humerus.

### Prognostic factors

There was a significant difference in overall survival between different age groups (*p* < 0.0001), Fig. 1c. Patients 70 years or older had a significantly worse overall survival rate than the other age groups together (*p* < 0.0001). In order to study how much higher age per se influenced the survival the Standardized mortality rates (SMR) were calculated. Patients 70 years or older had SMR 22.5 (*p* < .0001) and patients younger than 70 years SMR 4.2 (*p* = 0.0017). There was a significant difference comparing the SMR values (*p* = 0.014), indicating that age >70 years is a significant prognostic factor after correction for normal aging. Men and women had no significant difference in survival (80% vs 91% 5-year survival). Extracapsular tumor extension gave a worse survival rate than tumors limited to the lymph glands, but was not significant (*p* = 0.057), Fig. 1d. Regarding N stage, the overall 2-, 5- and 10-year survival rates for N1 disease were 92, 85, and 67%, respectively, 91, 91, and 79% for N2 disease, respectively, and 67, 50, and 50% for N3 disease, respectively (Fig. 1e). There was a significant difference in survival between patients with N1-, N2-, and N3-stage tumors (*p* = 0.037), and N3-stage disease had a significantly worse prognosis than N1 and N2 disease together (*p* = 0.010).

### HPV status

P16 staining of the tumors was possible for 59 of the 68 patients, and showed that 69% of the tumors were p16 positive, Table 1. P16-positive patients were 6 years younger than p16-negative patients, and the male dominance was larger for p16-negative patients than for p16-positive. N1-stage tumors were more common among p16-positive than p16-negative patients, while N2 was less common. Sixty-eight percent of the p16-positive patients were treated with ND and radiation compared to 44% for p16-negative patients.

The overall 2-, 5-, 10-year survival rates for p16-positive patients were 93, 88, and 82%, and for patients with p16-negative tumors, the survival rates were 67, 61, and 39%, respectively, Fig. 1f. The survival rates were significantly higher for patients with p16-positive tumors than with p16-negative tumors (*p* = 0.0005).

There was a significantly higher risk for recurrence among patients with p16-negative tumors than p16-positive tumors, 38% vs 4% (*p* = 0.0012), Fig. 1g.

### Treatment

In total, 43 patients were treated with unilateral neck dissection, Table 2. The majority had a modified radical ND with the accessory nerve preserved. Most patients, 53 of 61, were irradiated bilaterally to the neck (40.8 Gy to the contralateral side if free from disease, 64.6 if diseased). The majority, 57 of 61 patients, was irradiated to the pharynx to prevent an emergence of the primary tumor, but the nasopharynx was only irradiated in 15 patients (in patients with metastases in the retropharyngeal area or the posterior neck). Chemotherapy was given more often in combination with definitive radiotherapy (18 of 24 patients) than in combination with neck dissection and postoperative radiotherapy (6 of 36 patients).

**Table 2:** Patient, tumor, and treatment data divided by treatment

	Treatment A	Treatment B	*p*-value A vs B	Treatment C
Subjects, *n*	36	25		7
Age at diagnosis (years)				
Mean (SD)	56.6 (9.4)	61.2 (10.9)	0.12	67.1 (13.9)
Median (range)	57 (36–78)	56 (45–87)		63 (53–85)
Gender, male *n* (%)	32 (89)	20 (80)	0.55	3 (43)
Extracapsular extension				
Yes	11 (38)	0		0
No	18 (62)	8 (100)	0.081	4 (100)
p16 status				
Positive	28 (78)	10 (63)		3 (43)
Negative	8 (22)	6 (38)	0.42	4 (57)
N stage				
N1	5 (14)	6 (24)		2 (29)
N2	22 (61)	16 (64)		5 (71)
N2a	14 (39)	6 (24)		4 (57)
N2b	8 (22)	4 (16)		1 (14)
N2c	0	6 (24)		0
N3	9 (25)	3 (12)	0.84	0
Surgery				
RND	4 (11)	.		1 (14)
Modified RND	28 (78)	.		4 (57)
SOND	4 (11)	.		2 (29)
Radiotherapy				
Radiation schedule				
I	31 (86)	18 (75)		.
II	5 (14)	1 (4)		.
III	0	5 (21)	0.011	.
Radiation to the pharynx				
Pharynx incl. nasopharynx	3 (9)	10 (42)		.
Pharynx excl. nasopharynx	33 (91)	11 (46)		.
No radiation	0	3 (13)	0.0003	.
Radiation to the neck				
Bilateral	33 (91)	20 (83)		.
Ipsilateral	3 (9)	4 (17)	0.56	.
Chemotherapy	6 (17)	18 (72)	<0.0001	.

Treatment A, neck dissection and postoperative (chemo) radiation; Treatment B, (chemo) radiation, Treatment C, neck dissection. *SCC* squamous cell carcinoma, *RND* radical neck dissection, *SOND* supraomohyoidal neck dissection. Schedule I, hyperfractionated, accelerated radiotherapy to 64.6 Gy; Schedule II, slightly accelerated radiotherapy to 68 Gy; Schedule III, conventional radiotherapy to 66 Gy

Thirty-six patients were treated with Treatment A, 25 patients with Treatment B and seven patients with Treatment C, Table 2. Group C consisted of a small, older group than groups A and B, and four of the patients declined the planned postoperative radiation, which made comparisons involving this group uncertain.

Treatment A and Treatment B had similar age and gender distributions. The tumor histology was similar between Treatment A and Treatment B, and data regarding extracapsular extension was not available for most patients treated with primary (chemo) radiation. For patients treated with Treatment B, 24% had N1 disease vs 14% with Treatment A, and 12% had N3 disease vs 25%, but the differences were not significant. There was a significant difference regarding the radiation schedule used and radiation to the nasopharynx between groups A and B since they were used during different time periods. Significantly more patients had chemotherapy in the Treatment B group than in the Treatment A group, 72% vs 17% (*p* < 0.0001).

Patients treated with Treatment A and Treatment B had overall 2-, 5-, and 10-year survival rates of 86, 81, and 81%, respectively, vs 88, 88, and 76%, respectively (with no significant difference between Treatment A and B, Fig. 1h). The 2-, 5-, and 10-years survival for p16 positive patients for Treatment A were 93, 89, and 89% respectively, and for Treatment B 90%, 90%, and 80% respectively, with no significant difference between the groups (*p* = 0.24). The 2-, 5-, and 10-years survival for p16 negative patients for Treatment A were 63%, 50%, and 50% respectively, and for Treatment B 67%, 67%, and 50% respectively, with no significant difference between the groups (*p* = 0.80). The overall survival rate was significantly lower in the small Treatment C group compared to the other two treatments together (*p* = 0.0035).

There was no difference in recurrence between patients treated with Treatment A and Treatment B (2/36 patients, vs 1/25 patients). In Treatment C, the risk for recurrence was significantly higher in comparison to the other two groups together (5/7 patients, *p* < 0.0001).

### Multivariable analysis

Univariable analyses were performed regarding; overall survival and: −age, gender, N-stage, ECE, HPV status, and treatment. Multivariable analysis was performed with a forward stepwise Cox proportional hazard regression analysis of the survival of the four factors that were significant at univariable analysis: age > 70 years, N3 tumor, HPV status, and treatment. The independent predictors for survival were higher age (hazard ratio 2.73 per 10 year higher age, *p* < 0.0001), N3 stage (hazard ratio 6.25, *p* = 0.0013), and HPV status (hazard ratio 4.30, *p* = 0.0032).

## Discussion

The current study aimed to investigate the impact of prognostic factors for curatively treated HNCUP, including HPV status and survival depending on two different treatment modalities. Different possible prognostic factors were explored.

First, age was considered, and the material was divided into different age groups. Patients 70 years or older had a significantly worse prognosis than patients younger than 70 years, but there were no differences in survival between the other age groups. In previous studies low age was associated with significant improved survival. In one study the cut-point age was set to 62 years, in another 64 years, and in yet another 65 years [2–4]. The majority of patients in our study were males (81%), and males and females had similar survival rates, which has been seen in previous studies [2]. ECE was found in 27% of the tumors, which was slightly lower than in previous studies [3] but is uncertain due to a high proportion of missing data. Positive ECE was associated with lower survival, which has been shown in previous studies [3, 7, 8, 20, 21]. A majority had N2 tumors (63%), which was also the case in most previous studies, but the proportion of N3 tumors (18%) was relatively low [22]. Patients with N3 tumors had a significantly worse prognosis compared to N1 and N2 tumors together. N1 and N2 tumors, however, did have similar survival outcomes, as well as the subgroups of N2: N2a, N2b, and N2c. Some previous studies of N stage and HNCUP showed that N3 was significantly associated with worse survival [4, 8], and other studies showed that N2b, c and N3 had worse survival [7] or that N1, N2, N3 had decreasing survival [2, 3]. The results from the present study suggested that HNCUP patients with N3 tumors should be separated from the rest, being subject to a more aggressive treatment.

The majority of the tumors, 69%, were HPV positive by p16 immunostaining. The high p16 prevalence for HNCUP is not unexpected since HPV has been shown to be common in other head and neck cancers, especially tonsil and tongue base cancer [10]. One can even suggest that HNCUP could be considered a subtype of these cancers, since they are the most common occult primary, having cystic metastasis in the same area of the neck with similar age and prognostic panorama. The reported HPV prevalence for HNCUP differs widely in previous studies, between 22 and 91% [11, 12, 23, 24]. Larger studies are needed to better define the HPV prevalence and, it probably varies in different parts of the world and over time.

The overall survival was significantly higher and the risk for recurrence significantly lower for patients with p16-positive than p16-negative HNCUP. Other retrospective studies have also found better survival for HPV-positive HNCUP [23, 25, 26]. The positive prognostic effect of HPV is probably caused by a different and more favorable carcinogenic mechanism compared to cases with tobacco smoking as the main causal factor and in addition by the induction of an HPV-specific immune response [27, 28]. Since p16 is an important prognostic factor in HNCUP, one can suggest that p16 or other HPV analysis should be performed routinely. Additionally, there is a need for a prospective treatment study comparing HPV-positive and HPV-negative patients to investigate the optimal treatment. It was also interesting to observe the non-significant differences between the p16-positive patients, being 6 years younger with a higher proportion of females and more N1 tumors than the p16-negative patients. These disparities could probably be explained by differences in the features of the patients who are at risk for HPV infection vs smoking patients and differences in aggressiveness between the HPV- and tobacco-induced HNCUP. Possibly, the number of patients in this study was too low to show significant differences for these factors.

There was an opportunity to study the prognostic importance of the treatment for HNCUP since there was a change in treatment policy at our institution in the middle of the study period. Mainly, Treatment A (ND and radiation) was compared with Treatment B ([chemo] radiation). The overall 2-, 5-, and 10-year survival rates were similar after these two treatments. Previous studies on the treatment of HNCUP came to different conclusions: some studies found a statistically increased survival rate for treatment including ND [4, 8, 29]. In one study, the indication for ND was questioned since no significant difference in survival was found between the groups [14], and in other studies no statistically significant differences in survival between the treatment groups were reported, and no conclusions about the optimal treatment were drawn [2, 6, 13, 22, 30–32]. The comparison of treatments in the current study as well as in the previous studies was, however, limited by differences in some clinical and treatment factors. When comparing Treatment A and B for p16 positive patients, no significant difference in survival was observed, nor was there any significant difference for the p16 negative patients. No firm conclusions can be drawn from this study regarding the optimal treatment for HNCUP, although the treatment regimens used seemed to be comparable and with a good clinical outcome. The results of treatment with only neck dissection are difficult to interpret, but may be associated with an increased risk for recurrence.

The overall 5-year survival rate was 82%, which was high compared to most previous studies where survival rates ranged from 22 to 89% [2–5, 7, 8, 13–17, 20–22, 31, 33, 34]. Explanations for the good outcome could be the favorable status of different prognostic factors, including ECE, N stage, and HPV status. The administered radiation (90% were irradiated) was probably also critical for a favorable outcome. The radiation was given to a full dose of 64–68 Gy, the neck was irradiated bilaterally, and at least to an adjuvant dose if it was a unilateral known disease, and the mucosa of the pharynx (at least the oro-hypopharynx) was irradiated. A conclusion could be that these criteria should be fulfilled when administrating radiation for patients with HNCUP to achieve a high chance for survival. However, the risk for side effects must also be considered. The therapy used here was mainly well tolerated, but known side effects, such as dry mouth, dysphagia, osteoradionecrosis, and possible long-term development of secondary cancer in the irradiated area, were not recorded.

A limitation with the current study was the retrospective design resulting in the lack of some wanted information, for example smoking habits and performance status. During the rather long study period part of the diagnostic recommendations were changed. For example PET/CT and BOT biopsies were more frequently performed during the latter part of the study period, and today they are routinely used. Another limitation is the relatively small number of patients, however, most previous studies regarding HNCUP comprised an even lower number of patients, and no prospective study has been performed probably because HNCUP is rare. For the HPV analysis, only p16 immunostaining was used. In some other studies, DNA-techniques were also used to determine HPV status. We did not have access to those techniques. On the other hand, p16 staining is an inexpensive, reliable, and accessible tool that can be used in routine clinical practice as part of the treatment plan.

## Conclusions

HPV is common in curatively treated HNCUP, and 69% of the tumors were HPV positive by p16 immunostaining. P16 positivity is associated with significantly higher survival and a significantly decreased risk for tumor recurrence; the 5-year overall survival rates for patients with p16-positive tumors were 88% vs 61% for p16-negative patients. N3 tumors and age over 70 years were significant negative prognostic factors. Treatment with neck dissection and postoperative radiation or (chemo) radiation both gave favorable outcomes with similar results. HNCUP has a good prognosis, with an overall 5-year survival rate among the curatively treated patients of 82%.

## Abbreviations

ECE: Extracapsular extension
HNCUP: Head and neck cancer from an unknown primary
HPV: Human papilloma virus
ICD-10: International Classification of Diseases-10
IMRT: Intensity-modulated radiotherapy
ND: Neck dissection
RT: Radiotherapy
